# Poisoning by Opium of a Suckling Babe through Its Mother

**Published:** 1875-09

**Authors:** 


*Poisoning by Opium of a Suckling Babe through its Mother.*—The *Boston Medical and Surgical Journal* says: "In our foreign exchanges we learn of a case of poisoning of an infant by opium administered to the mother. The latter was about to undergo an operation, and at ten o'clock in the morning she took twenty-five drops of Battley's sedative solution, and repeated the dose at two o'clock. At eight o'clock in the evening she took five centigrammes of opium in a pill.

"Her child, a strong boy seven weeks old, was restless throughout the day. At midnight he took the breast, and suddenly fell into a deep sleep, in which he remained for six hours. On awaking he sucked a little, and again slept throughout the day. At two P. M. respiration diminished in frequency, and became less deep and jerking. At six P. M. the pupil was contracted, respiration imperfect, jerking irregular, but in frequency nearly normal. It was with great difficulty that he could be aroused. Coffee was administered by the mouth and by the rectum, and the patient was exposed to the draught from an opon window, and in about an hour he seemed better. An hour later respiration ceased for a while, and he appeared dead; life, however, returned, and the following day by two A. M. he was out of danger."—*Medical Times.*

